# Reliability of a VO_2_-derived running-speed threshold in trained men

**DOI:** 10.1080/15502783.2025.2550179

**Published:** 2025-08-22

**Authors:** Marialaura Espaillat, David M. Falkins, Brandi Antonio, David H. Fukuda, Jeffrey R. Stout

**Affiliations:** University of Central Florida, Physiology of Work and Exercise Response (POWER) Lab, Institute of Exercise Physiology and Rehabilitation Sciences, Orlando, FL, USA

**Keywords:** GXT, heart rate, running, threshold

## Abstract

**Background:**

This study aimed to establish the test-retest reliability of a VO_2_-derived running-speed threshold (RSVO_2_) in trained men.

**Methods:**

Fourteen aerobically trained males (20.6 ± 1.9 y; 69.4 ± 10.2 kg; 176.1 ± 7.1 cm; VO₂peak 50.5 ± 7.8 mL·kg^−1^ ·min^−1^; time-to-exhaustion 27.5 ± 6.9 min) performed a graded-exercise test (start = 8 km·h^−1^, 0% grade; +1 km·h^−1^ every 3 min) to establish VO₂peak. During two subsequent visits, they completed four 8-min steady-state runs at 60, 70, 80, and 90% of final GXT speed, presented in random order. Minute-by-minute VO₂ from minutes 4–8 was averaged. The rate of VO₂ rise (ΔVO₂/Δt) was regressed against speed; the y-intercept at slope = 0 defined RSVO₂. Reliability statistics included ICC(2,1), standard error of measurement (SEM), coefficient of variation (CV), and minimal detectable change at 95% confidence (MDC95).

**Results:**

RSVO₂ showed excellent reliability: ICC(2,1) = 0.96 (95% CI 0.89–0.99); SEM = 0.35 km·h^−1^; CV = 3.0%; MDC95 = 0.98 km·h^−1^. Threshold values did not differ between visits (*p* = 0.33). RSVO_2_ thresholds occurred at an average of 72.6% of the top stage completed during the GXT.

**Conclusions:**

An eight-minute, multi-intensity protocol yields a highly reliable VO₂-based running-speed threshold in trained men. Future research should validate RSVO₂ against ventilatory and lactate thresholds and examine its applicability across sexes and clinical populations.

